# Prevalence of Hypertension in Adolescents: Differences Between 2016 ESH and 2017 AAP Guidelines

**DOI:** 10.3390/jcm14061911

**Published:** 2025-03-12

**Authors:** Caterina Carollo, Luigi Peritore, Alessandra Sorce, Emanuele Cirafici, Miriam Bennici, Luca Tortorici, Riccardo Polosa, Giuseppe Mulè, Giulio Geraci

**Affiliations:** 1 Unit of Nephrology and Dialysis, Hypertension Excellence Centre, Department of Health Promotion, Mother and Child Care, Internal Medicine and Medical Specialties (PROMISE), University of Palermo, 90133 Palermo, Italy; caterina.carollo@unipa.it (C.C.); alessandra.sorce@community.unipa.it (A.S.); miriam.bennici@community.unipa.it (M.B.); luca.tortorici@unipa.it (L.T.); giuseppe.mule@unipa.it (G.M.); 2 Unit of Nephrology and Dialysis, Department of Clinical and Experimental Medicine, University of Messina, 98125 Messina, Italy; 3 Department of Medicine and Surgery, “Kore” University of Enna, 94100 Enna, Italy; polosa@unict.it (R.P.); giulio.geraci@unikore.it (G.G.); 4 Center of Excellence for the Acceleration of Harm Reduction, University of Catania, 95124 Catania, Italy

**Keywords:** hypertension, juvenile hypertension, adolescents, HT Guidelines

## Abstract

**Introduction:** The American Academy of Pediatrics (AAP) published in 2017 new guidelines for the screening and management of hypertension in children containing different nomograms compared to the European guidelines, leading to a reclassification of blood pressure values, the consequences of which are still little investigated. The aim of our study was to evaluate the prevalence of high blood pressure values estimated with both the most recent American and European guidelines and to analyze the relationship of blood pressure increases with lifestyles and potentially risky behaviors in a school population in Western Sicily. **Methods:** On the occasion of the XV World Hypertension Day, blood pressure values of 1301 students aged between 13 and 18 were measured. Two questionnaires were administered, one relating to anamnestic data and anthropometric parameters and a second aimed at investigating lifestyle. For the diagnosis of increased blood pressure, both ESH and AAP criteria were considered. **Results:** The prevalence of elevated blood pressure was 7.5% according to ESH criteria and nearly twice as high using AAP criteria, with a more pronounced discrepancy in females. Individuals with elevated blood pressure were younger, exhibited higher body weight and BMI, and had an increased prevalence of overweight and obesity. Classification based on ESH criteria revealed higher alcohol and drug consumption among normotensive individuals. AAP criteria identified a higher proportion of males and greater height in the hypertensive group. Systolic blood pressure correlated significantly with height, weight, and BMI, with stronger associations in males, while diastolic pressure correlated with weight and BMI. **Conclusions:** To the best of our knowledge, our study is the only one to analyze the prevalence of increased blood pressure and its relationship with lifestyle factors and anthropometric data in adolescence in our region. Our study confirms that elevated blood pressure is common in adolescence, with higher prevalence using the 2017 AAP criteria than ESH guidelines.

## 1. Introduction

In recent years, the notion that primary or essential hypertension is not a rare condition in the pediatric population has become well established. It is now recognized as the most prevalent form of hypertension in this age group, with both its incidence and prevalence showing an upward trend.

The prevalence of arterial hypertension in pediatric populations, particularly during adolescence, is increasingly recognized as showing an upward trend, a view supported by the majority of experts. It also seems equally certain that this rise cannot be solely attributed to the more frequent measurement of blood pressure. This increase is largely attributed to the “explosion” of obesity among children and adolescents in economically developed countries [1,2,3], as well as the growing prevalence of unhealthy lifestyle habits (sedentary behavior, insufficient physical activity, excessive intake of salty foods, junk food, and excessive use of video games and smartphones). Early obesity leads to metabolic dysregulation and vascular changes that predispose individuals to atherosclerosis.

Adolescent hypertension further exacerbates these alterations, increasing the likelihood of adverse cardiovascular outcomes later in life. The definition of normal blood pressure values in pediatric patients is complicated by the close correlation between blood pressure and growth parameters, such as height and weight. Consequently, it is essential to establish normative criteria based on age- and height-specific percentiles, which should ideally be derived from data collected in the relevant geographical populations. However, in the last years, even in Italy, the percentiles developed from North American pediatric populations have been employed. More recently, the European Society of Hypertension (2016) and the American Academy of Pediatrics (AAP) (2017) released updated guidelines for the screening and management of hypertension in children and adolescents [4,5]. These guidelines differ not only from one another but also from the American guidelines established in 2004, leading to considerable uncertainty and confusion among pediatricians and healthcare professionals managing hypertensive children. Few studies have systematically compared the effects of reclassifying blood pressure values based on the application of these two distinct sets of guidelines, particularly within the adolescent population.

The aim of our study was to assess the prevalence of elevated blood pressure values as estimated according to the most recent American and European guidelines, and to analyze the relationship between blood pressure increases and lifestyle factors and potentially risky behaviors in adolescents from a school-based population in Western Sicily.

## 2. Materials and Methods

This study was conducted using data collected during the XV World Hypertension Day in May 2019, which involved measuring blood pressure values and interviewing students from seven secondary schools in Western Sicily. After obtaining written informed consent from the parents for the evaluation of their children, 1301 students aged between 13 and 20 years, enrolled in the aforementioned schools, were recruited. The students were administered a questionnaire consisting of questions related to their medical history and anthropometric parameters, a tool that has been used for several years during World Hypertension Day events (File S1). Additionally, a second, more comprehensive questionnaire was provided, partly based on the one used in the I-Game study (a multicenter observational study proposed by the Italian Society of Hypertension (SIIA) aimed at assessing risk factors for juvenile hypertension) [6]. This second questionnaire gathered information on family history of hypertension, cardiovascular-, renal-, and cancer-related diseases, birth weight, dietary habits, frequency of smartphone and computer usage, consumption of coffee, drugs, alcohol, and tobacco, as well as physical activity (File S2). Blood pressure measurements were taken at the school premises, and the questionnaires were administered anonymously to ensure greater comfort when answering “difficult” questions, such as those regarding alcohol and drug consumption. The language used in the questionnaire was simple and age-appropriate for the participants. All participants underwent blood pressure measurement using one of two validated oscillometric devices (Omron M6 Comfort and Microlife BPA150). Three consecutive BP measurements at 1 min intervals were collected after a five minute rest in the sitting position, with the back and arm supported and feet flat on floor, and the average of the 2nd and 3rd measurements was recorded. Pulse rates were also automatically displayed by the device and recorded. To diagnose an increase in blood pressure values, we applied both the ESH and AAP criteria (Table 1). According to all international guidelines, a single blood pressure measurement does not reliably define the presence of hypertension. Hypertension can only be diagnosed, as per both current and previous European and U.S. guidelines, when elevated blood pressure is confirmed on at least three separate occasions. Therefore, we prefer not to label the detection of blood pressure values exceeding the normal ranges outlined by the aforementioned guidelines as “hypertension”. Instead, we will refer to this condition as “increased blood pressure”.

For the definition of height percentiles, which are essential for determining normal blood pressure thresholds in students under 16 years old according to European guidelines, and in those under 13 years old according to North American guidelines, we used the WHO growth tables [7].

To define overweight and obesity, we calculated the body mass index (BMI) using the standard formula (Weight/Height^2^), based on the anthropometric measurements provided in the students’ questionnaires. The resulting BMI value was then used to calculate the BMI Z-score for each individual, using the formula (Xn − µ)**/**σ, where Xn represents the individual BMI value, µ is the mean BMI of the entire population, and σ is the corresponding standard deviation, applying the criteria suggested by the WHO in 2007. The Z-score measures how many standard deviations each sample value deviates from the mean.

In accordance with de Onis et al. [7], individuals with a BMI Z-score ≥ 1.04 (or ≥85th percentile) were classified as overweight, and those with a BMI Z-score ≥ 1.64 (or ≥95th percentile) were considered obese. The decision to use WHO criteria aligns with recommendations from consensus by the Italian Society of Pediatrics and the Italian Society of Pediatric Endocrinology and Diabetes [8]. This approach is based on the need to offer a system that, while not ideal for comparing the growth of individual children or groups, demonstrates greater sensitivity in identifying overweight and obese children and adolescents compared to older Italian criteria, likely due to the fact that the measurements underlying the WHO criteria were taken following the epidemic increase in obesity.

### Statistical Analysis

Statistical analysis was performed using the IBM-SPSS version 26 software package. Categorical variables were described in terms of absolute frequency and percentage, while continuous variables were further subdivided into two groups based on the results of the Kolmogorov–Smirnov test. Normally distributed variables were expressed as the mean ± standard deviation, whereas non-Gaussian variables were expressed as median and interquartile range.

Where applicable, variables characterized by extreme skewness or kurtosis were normalized using the z-score. The study population was initially divided into two distinct groups according to the criteria of the European Society of Hypertension and the American Academy of Pediatrics guidelines. The population was subsequently further subdivided based on the presence or absence of elevated blood pressure, as defined by both sets of guidelines.

Differences in demographic, anthropometric, clinical, blood pressure, and lifestyle-related data between the two groups were evaluated using the Student’s *t*-test for normally distributed continuous variables. To assess differences in categorical data proportions between the two guidelines, the McNemar test was employed. Effect sizes (Cohen’s d for continuous variables and odds ratios for categorical variables) and 95% confidence intervals were calculated. In the analysis of correlations between variables of interest, Pearson’s correlation coefficient was computed, taking into account the data distribution.

Subsequently, multivariate linear regression models were used for the significant relationships. The null hypothesis was rejected in all two-tailed tests for *p*-values < 0.05.

## 3. Results

**Figure 1:**
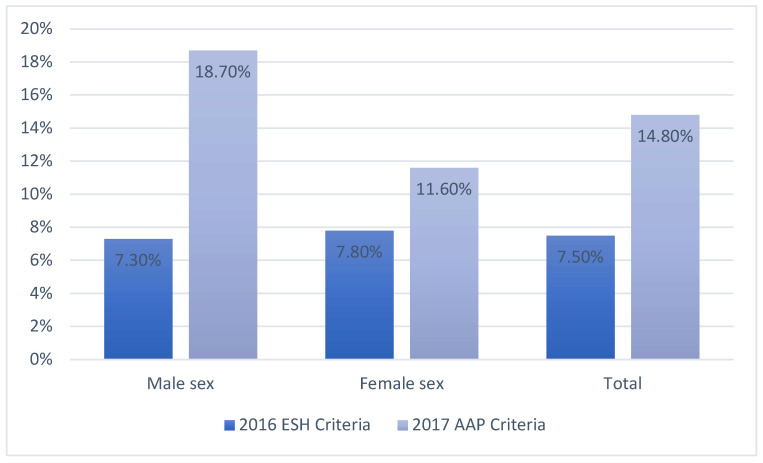
Prevalence of elevated blood pressure according to the 2017 AAP and the 2016 ESH guidelines.

The prevalence of increased blood pressure was 7.5% when assessed using the ESH classification criteria and almost twice as high when the AAP criteria were applied (Figure 1). This discrepancy was more pronounced in females than in males.

Table 2 presents the main demographic and anthropometric characteristics of the entire study group, as well as the two subgroups with and without elevated blood pressure, classified according to the threshold values outlined in the aforementioned guidelines.

It can be observed that individuals with blood pressure above the normal range are younger and have a higher body weight and BMI and, consequently, a higher prevalence of overweight and obesity compared to those with normal blood pressure, regardless of the guidelines used for categorization. A higher prevalence of males and greater height in the group with elevated blood pressure is observed only when using the AAP criteria.

Individuals with higher systolic blood pressure (SBP) and diastolic blood pressure (DBP) also exhibit a higher heart rate, regardless of the criteria used to classify the population (Table 3).

Regarding lifestyle factors, differences emerged only when using the ESH guidelines to classify the population, specifically a higher consumption of alcoholic beverages and drugs (the latter at the borderline of statistical significance) among individuals with normal blood pressure. Additionally, within this group, there was a tendency (also with borderline significance) for a higher percentage of students engaging in regular physical activity (Table 4).

The analysis of dietary habits revealed significant differences in the prevalence of elevated blood pressure between the two guidelines (Table 5), indicating that AAP 2017 identified a significantly higher proportion of adolescents with elevated blood pressure, particularly in relation to junk food consumption (*p* < 0.0001) and high meat intake (*p* = 0.02). Conversely, ESH 2016 showed higher proportions of elevated blood pressure associated with low fruit and vegetable intake (*p* = 0.03) and excessive screen time (*p* = 0.05).

**Figure 2:**
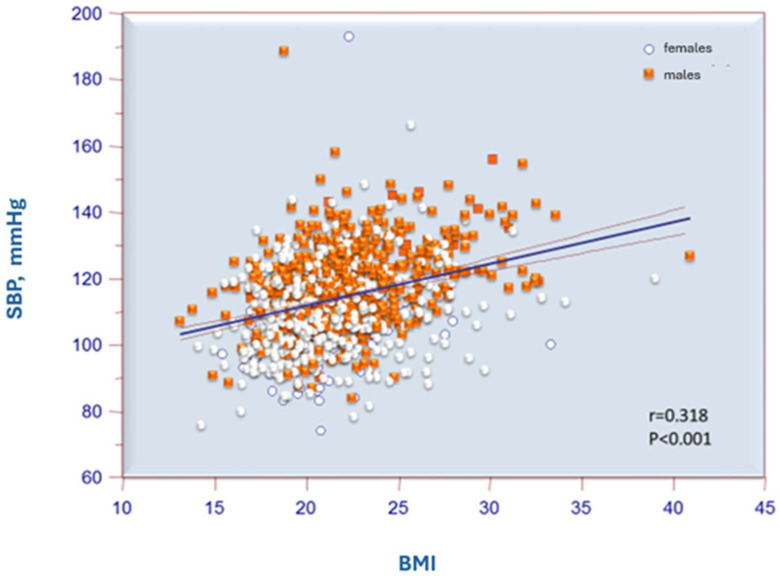
Correlation between systolic blood pressure and BMI.

Table 6 shows the correlations between blood pressure values and anthropometric variables in the total population and by sex. Statistically significant correlations were found between systolic blood pressure and height, weight, and BMI (Figure 2). It is noticeable that the correlations of these parameters are stronger in males compared to females. Diastolic blood pressure values, on the other hand, showed significant correlations only with weight and BMI.

Both guidelines suggest that infrequent fruit consumption (less than twice per day) is associated with an increased risk of hypertension. However, the confidence intervals for both guidelines are wide, particularly for ESH 2016, indicating a lack of statistical significance. This suggests that while the association seems plausible, it is not conclusive and may not be a reliable predictor of hypertension. The AAP 2017 guideline presents a broader CI but still suggests a potential risk factor. Therefore, the ability of these guidelines to effectively predict hypertension risk based on fruit consumption is questionable.

**Figure 3:**
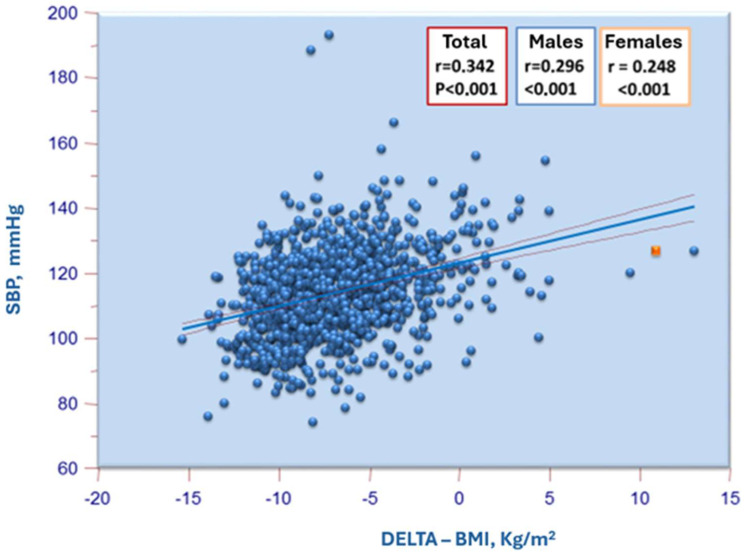
Correlation of systolic blood pressure with the difference (delta) between the observed BMI and the diagnostic BMI cut-off for obesity suggested by WHO (specific for age and sex).

In addition to evaluating the correlations between BMI and blood pressure values, we also correlated the latter, particularly systolic blood pressure, with the difference between the observed BMI value and the diagnostic BMI obesity cut-off suggested by the WHO (specific to age and sex). This was considered given that the thresholds for defining obesity and overweight in adolescents differ from those in adults and vary according to the demographic characteristics of the individuals examined. The association between systolic blood pressure and this body size index was found to be as significant, if not more so, than the relationship between the observed BMI and systolic blood pressure (Figure 3).

## 4. Discussion

Our study, conducted on a sample of adolescents attending some secondary schools in Western Sicily, stems from the desire to assess the distribution of blood pressure values and determine the differences in the prevalence of various blood pressure categories identified according to the new American and European guidelines, as well as to analyze the relationships with medical history data and lifestyle factors.

A key finding is the higher prevalence of elevated blood pressure values when using the new 2017 AAP criteria compared to the ESH guidelines. This result was widely anticipated, given that the American guidelines for adolescents over the age of 13 suggest abandoning the percentile method for diagnosing hypertension and using the same threshold values as adults, which were lowered to 130/80 mmHg in the 2017 guidelines of the American Heart Association and the American College of Cardiology [9]. In contrast, the ESH guidelines for pediatric hypertension management start equating blood pressure reference values to those of adults only at the age of 16, and the adult diagnostic cut-off has remained at the traditional 140/90 mmHg [10]. As a result, it is not uncommon to find individuals identified as hypertensive based on the American classifications who are not classified as such according to European criteria.

The prevalence found in our study was 7.5% when applying the European guidelines and almost double (14.8%) when using the AAP criteria. This discrepancy was also observed to varying degrees in other studies dedicated to this comparison and was confirmed by a meta-analysis conducted by Goulas et al. in 2021 [11]. This meta-analysis evaluates differences in hypertension classification, prevalence rates, and their impact on identifying LVH, a key marker of target organ damage. The findings suggest that the 2017 AAP guidelines, which use lower blood pressure thresholds and updated normative data, identify a higher prevalence of hypertension compared to the Fourth Report and ESH criteria. Consequently, this may lead to earlier intervention but also raises concerns about potential overtreatment. Additionally, the study examines how each guideline’s diagnostic criteria influence the detection of LVH, providing insights into the clinical implications of varying hypertension definitions in pediatric populations.

In the CARITALY study by Di Bonito et al. [12], the authors found a 4.1% increase when using the AAP 2017 guidelines compared to the ESH 2016 guidelines, and then, the study by Lurbe et al. [13] showed a prevalence of 6.6% according to the ESH guidelines versus 10.5% with the AAP guidelines.

More recently, Fanelli and colleagues [14], in a cohort of 624 adolescents, reported a prevalence of elevated blood pressure values of 32.7% according to the 2017 AAP guideline cut-offs, compared to only 17.6% based on the 2016 ESH definition. This difference was statistically significant (*p* < 0.01). The use of the AAP nomograms consistently identified a higher proportion of hypertensive individuals across all subgroups stratified by age, sex, and BMI.

In a retrospective analysis of 395 adolescents aged 13 to 16, Aksoy et al. evaluated both office and 24 h ambulatory blood pressure measurements, categorizing participants into three subgroups based on body mass index (BMI). Unlike the previously mentioned studies, the authors found that, across the entire cohort, the prevalence of hypertension was 32.4% according to the ESH GL2016 and 34.4% according to AAPG2017. Among obese adolescents, these rates increased to 38.8% and 43.3%, respectively. The diagnosis of hypertension was confirmed using both guidelines, with substantial agreement observed in both the obese subgroup and the overall study population, as indicated by kappa values of 0.738 and 0.785, respectively [15].

In our study, the correlations between systolic blood pressure values and anthropometric variables that were statistically significant included weight (r = 0.432), height (r = 0.321), and BMI (r = 0.318) in the total population. When differentiated by sex, the correlation indices were r = 0.398, r = 0.233, and r = 0.338 for males and r = 0.256, r = 0.089, and r = 0.226 for females. Diastolic blood pressure values, on the other hand, showed significant correlations only with weight and BMI. The findings confirm what has been highlighted in the studies previously cited.

The prevalence of obesity and overweight among the participants in our study was 22%. Obesity is increasing at an alarming rate among children and adolescents in all developed societies, with the United States leading the way, as they do with other aberrant behaviors [16]. Unfortunately, childhood obesity closely correlates with adult obesity [17], laying the foundation for all other consequences. An increase in abdominal circumference in school-aged children, measured at the abdomen, is a particularly unfavorable predictor of metabolic syndrome, which now affects most adults in the United States [18]. The causes of increased obesity in children and adolescents are due both to the increased caloric intake from the ever-growing number of fast food outlets [19] and, perhaps even more importantly, to the reduction in physical activity. The American Academy of Pediatrics has officially called for reduced access to canned drinks in schools [20], but this action alone will not be sufficient unless it is accompanied by measures addressing other involved factors [21]. Most notably, as a consequence of the rise in obesity, mean blood pressure in children and adolescents in the U.S. increased by 1.4/3.3 mmHg from 1990 to 2000 [22], which corresponds to significant increases in childhood hypertension, with higher likelihoods of hypertension in adulthood. These overweight children with hypertension may also experience more severe cardiovascular disease, as reflected in surrogate markers such as dyslipidemia [23], increased left ventricular mass, and carotid intima-media thickness [24]. The combination of obesity, increased blood pressure, and other cardiovascular risk factors has led to the prediction of a cardiovascular disease epidemic in adults in the near future [25]. Furthermore, obese children have been found to have an increased frequency of breathing disorders and sleep apnea [26], which can, as in adults, increase blood pressure. These children may experience less frequent sleep apnea than adults, usually exhibiting tachypnea and increased dyspnea [27].

In our study, we observed a prevalence of obesity and overweight, when considered together, of 22%; this figure is very similar to the results from the study by Buscemi et al. [28], which found a prevalence of 21.6% in a population of 478 teenagers. BMI in our study showed a stronger correlation with systolic blood pressure than with diastolic blood pressure, the latter not being statistically significant in females. It is likely that the greater impact of BMI on systolic blood pressure is related to the close relationship between body mass and cardiac output, which is one of the main determinants of systolic blood pressure.

The exact pathophysiological mechanism by which obesity causes hypertension is not well understood. The obesity–hypertension link is a complex, multifactorial condition that seems to involve insulin resistance, hyperactivation of the sympathetic nervous system, the renin–angiotensin system, abnormal renal sodium retention, possible leptin resistance, altered vascular reactivity, and changes in the hypothalamic–pituitary–adrenal axis. Many authors believe that fluid retention is the common factor linking obesity to hypertension. It seems that endogenous hyperinsulinemia in obese children following glucose load induces urinary sodium retention [29]. Another hypothesis is that of insulin receptor structure alteration or a defect in the target tissue [30]. Finally, structural changes in the kidneys of obese individuals may cause fluid retention. Insulin resistance seems to be the metabolic link connecting obesity to hypertension. Children with borderline blood pressure have higher plasma insulin levels and greater weight than normotensive individuals. The renin–angiotensin–aldosterone system plays a crucial role in regulating the tone of the glomerular efferent arteries and sodium reabsorption. Its activity is modulated by salt intake and blood pressure.

Rocchini et al. [29] observed that an increase in plasma renin activity caused a greater increase in aldosterone concentration in obese adolescents compared to normal-weight controls. Regarding the SNS, it is thought that in obese individuals, it is chronically activated in an attempt to prevent further weight gain, and that hypertension is a byproduct of an overactive SNS [31]. The Bogalusa Heart Study reports that in a group of children, resting heart rate correlated positively with blood pressure and subscapular skinfold thickness [32].

The particular attention to this phenomenon is justified by the evidence that the combination of obesity and hypertension creates a double burden on the heart, ultimately leading to reduced ventricular function [33,34], which is why the importance of diagnosis and management in this age group is recognized. Hypertension not only affects the heart but also the kidneys, CNS, and blood vessels, causing early functional and structural changes that can be significant.

Therefore, early organ damage, as a measure of the clinical consequences of increased blood pressure, is essential for managing hypertension in youth. Monitoring organ damage in children and adolescents with high blood pressure is even more important because the cardiovascular/renal sequelae of hypertension that begins in childhood may not become clinically relevant until adulthood [35,36].

Regarding dietary habits, AAP 2017 associates junk food consumption and high meat intake more strongly with elevated blood pressure, which may lead to a larger number of diagnoses. In contrast, ESH 2016 appears to be more selective, diagnosing elevated blood pressure in a smaller group, possibly due to its emphasis on other dietary factors such as low fruit and vegetable consumption and excessive screen time. The higher number of cases with elevated blood pressure identified by AAP 2017 reflects a broader diagnostic approach, possibly including individuals with less severe elevated blood pressure or borderline cases, while ESH 2016 seems to focus on more clear-cut cases.

The guidelines of the American Academy of Pediatrics (AAP) and the European Society of Hypertension (ESH) emphasize that elevated blood pressure values in children and adolescents require a targeted therapeutic approach to prevent long-term cardiovascular risk. A key aspect is the shift in terminology from “hypertension” to “elevated blood pressure values”, aiming to reduce the risk of overtreatment and avoid unnecessary pharmacological interventions in cases where blood pressure is only slightly above normal limits. The first-line intervention remains lifestyle modifications, including a balanced diet, sodium intake reduction, regular physical activity, and weight management. In cases of persistent or secondary hypertension, pharmacological therapy may be indicated, with agents such as ACE inhibitors, angiotensin receptor blockers, calcium channel blockers, or beta-blockers selected based on the underlying condition and patient tolerance. Regular blood pressure monitoring is essential to optimize therapeutic management and prevent future complications.

The results of our study should be interpreted with caution, considering some of its methodological limitations. One of the main limitations is the inability to diagnose hypertension definitively, as blood pressure values were measured only once. Blood pressure measurement, according to all international guidelines, when taken on a single occasion, cannot reliably define the presence of hypertension. Indeed, this diagnosis can only be made, according to both the ESH and AAP guidelines, when elevated blood pressure levels are confirmed on at least three different occasions. Therefore, as mentioned earlier, we preferred not to use the term “hypertension” to define cases where blood pressure values exceeded the normal ranges set by the aforementioned guidelines. Instead, we identified such cases as “increased blood pressure”. However, it is worth noting that in most pediatric hypertension screening studies, as in our study, blood pressure values have been measured on a single occasion. In the Di Bonito study [12], where blood pressure was measured only once, increased blood pressure was defined as a “high-risk hypertension condition”; in the study by Lurbe et al. [13], only part of the studied population received a formal diagnosis of hypertension based on repeated measurements at three different times and sometimes with the help of 24 h blood pressure monitoring.

Another significant limitation of our study is the use of self-reported data, particularly anthropometric measurements, and the use of questionnaires that did not guarantee anonymity for the collection of medical history and lifestyle data, with the greater risk of frequent misperceptions of body size, especially among females. This method of data collection, while necessary for practical purposes, exposes this study to the risk of inaccurate information. However, even with self-reported anthropometric data, the prevalence results are similar to those from the ABCD study [28], where such data were measured directly. A recent systematic review aimed at summarizing and evaluating the comparative validity of measured and self-reported height and weight data, as well as recommending strategies to improve the reliability of self-reported data collection in studies, revealed a good agreement between measured and self-reported values based on the intraclass correlation coefficient and Bland–Altman plots. However, due to biases such as social pressure and self-esteem issues, women tended to underestimate their weight, while men overestimated their height. Overall, self-reported measurements remain valuable indicators for supplementing limited direct anthropometric data, particularly in large-scale surveys [36].

On the other hand, our study, to the best of our knowledge, is the only one to analyze the prevalence of increased blood pressure and its relationship with lifestyle factors and anthropometric data in adolescence in our region. We aim not only to increase the sample size but also to confirm the diagnosis of hypertension in individuals who showed an increase in blood pressure and to objectively assess the self-reported risk factors and examine other parameters (e.g., uric acid), which were not assessed in the present study.

## 5. Conclusions

Despite the methodological limitations previously described, our study has confirmed that elevated blood pressure is far from rare during adolescence. This finding underscores the need for greater attention to this issue, recognizing that non-pharmacological interventions can prevent the onset of serious complications in adulthood.

## Figures and Tables

**Table 1:** Diagnostic criteria of increased blood pressure according to the 2017 AAP guidelines compared with the 2016 ESH guidelines.

	2017. AAP GL		2016. ESH GL	
	<13 Years	>13 Years	<16 Years	>16 Years
Increased blood pressure	From ≤90th to <95th percentile or 120–129/<80	120–129/<80	From ≥90th to <95th percentile	130–139/85–89

**Table 2:** Demographic and anthropometric characteristics of the study population and the two subgroups classified according to the ESH and the AAP Criteria.

	2016 ESH Criteria			2017 AAP Criteria		
	Increased BP (*n* = 99)	Normal BP (*n* = 1202)	*p*	Increased BP (*n* = 195)	Normal BP (*n* = 1122)	*p*
Age (years)	15.6 ± 1.7	16.6 ± 1.55	<0.001 −0.65 [−0.84–0.46]	16.8 ± 1.7	16.5 ± 1.6	0.006 0.18 [0.04–0.32]
Male sex *n* (%)	46 (46)	541 (45)	0.753 1.03 [0.67–1.58]	110 (56)	477 (43)	<0.001 1.64 [1.20–2.25]
Height (cm)	168.9 ± 9.6	168.7 ± 9.1	0.443 0.02 [−0.18–0.22]	171 ± 9.3	168.2 ± 9	<0.001 0.30 [0.14–0.46]
Weight (kg)	65.7 ± 13.8	61.3 ± 11.8	<0.001 0.34 [0.15–0.53]	69.2 ± 14	60.3 ± 11	<0.001 0.70 [0.54–0.86]
BMI (kg/m^2^)	23 ± 3.4	21.5 ± 3.2	<0.001 0.45 [0.26–0.64]	23.4 ± 3.7	21.2 ± 3	<0.001 0.64 [0.45–0.83]
Overweight, *n* (%)	39 (39.4)	202 (16.8)	<0.001 3.18 [2.12–4.77]	66 (33.8)	175 (15.5)	<0.001 2.77 [1.95–3.93]
Obesity, *n* (%)	11 (11.1)	35 (2.9)	<0.001 4.21 [2.07–8.58]	23 (11.8)	23 (2)	<0.001 6.60 [2.42–12.75]

**Table 3:** Blood pressure values and heart rate of the study population and the two subgroups classified according to the ESH and the AAP Criteria.

	2016 ESH Criteria			2017 AAP Criteria		
	Increased BP (*n* = 99)	Normal BP (*n* = 1202)	*p*	Increased BP (*n* = 195)	Normal BP (*n* = 1106)	*p*
SBP (mmHg)	132 ± 15	112 ± 11	<0.001 1.53 [1.33–1.73]	130 ± 12	111 ± 10	<0.001 0.18 [0.04–0.32]
DBP (mmHg)	83 ± 34	67 ± 7	<0.001 0.62 [0.42–0.82]	81 ± 25	66 ± 6	<0.001 0.30 [0.14–0.26]
HR (bpm)	88 ± 18	83 ± 20	0.011 0.02 [−0.18–0.22]	89 ± 18	82 ± 21	<0.001 0.67 [0.47–0.87]

Abbreviation: (SBP = systolic blood pressure, DBP = diastolic blood pressure, and HR = heart rate).

**Table 4:** Prevalence of lifestyle habits and regular physical activity, as reported in questionnaires administered to students, in the entire study population and in the two subgroups defined according to the ESH and AAP criteria.

	Total	Increased BP	Normal BP	*p*	Increased BP	Normal BP	*p*
		2016 ESH			2017 AAP		
Current smokers, *n* (%)	203 (15)	13 (13)	190 (16)	0.56 0.71 [0.39–1.30]	35 (18)	168 (15)	0.284 1.31 [0.88–1.95]
Alcohol consumption, *n* (%)	267 (21)	9 (9)	258 (21)	0.03 0.36 [0.18–0.73]	42 (25)	227(20)	0.7 1.44 [0.99–2.11]
Drugs consumption, *n* (%)	88 (7)	2 (2)	86 (7)	0.058 0.26 [0.06–1.09]	17 (9)	71 (6)	0.2 1.48 [0.85–2.57]
Regular physical activity, *n* (%)	192 (15)	8 (8)	185 (15)	0.055 0.48 [0.23–1.00]	34(17)	163 (15)	0.32 1.30 [0.87–1.95]

**Table 5:** Prevalence of dietary habits and screen time, as reported in questionnaires administered to students, in the entire study population and in the two subgroups defined according to the ESH and AAP criteria.

	2016 ESH		2017 AAP		
Dietary Habits and Screen Time	%	OR, 95% CI	%	OR, 95% CI	*p*
Fruit < 2 times/day (%)	36. 8	[0.3684 (0.1224–1.1092)]	27.1	[2.7143 (0.9015–8.1722)]	0.02
Vegetables < 3 times/day (%)	69.2	[0.692 (0.3612–1.3257)]	44	[1.4452 (0.7543–2.7686)]	0.03
Junk Food > 1 time/week (%)	27	[0.3597 (0.2159–0.5991)]	35	[2.7802 (1.6691–4.6309)]	0.0001
Meat consumption > 2 times/day (%)	28.2	[0.5652 (0.2662–1.2)]	41%	[1.1579 (0.2203–6.0849)]	0.02
Screen Time > 2 h\die (%)	55	[1.5504 (0.7856–3.0598)]	26%	[0.645 (0.3268–1.273)]	0.05

**Table 6:** Pearson correlation coefficients of SBP and DBP with selected variables examined in the entire study population and stratified by gender.

		Total		Males		Females	
		SBP	DBP	SBP	DBP	SBP	DBP
Height. cm	r	0.321	0.02	0.233	0.038	0.089	0.039
	*p*	<0.001	0.47	<0.001	0.362	0.018	0.305
Weight. kg	r	0.432	0.102	0.398	0.15	0.256	0.099
	*p*	<0.001	<0.001	<0.001	<0.001	<0.001	0.009
BMI	r	0.318	0.116	0.338	0.157	0.226	0.087
	*p*	<0.001	<0.001	<0.001	<0.001	<0.001	0.023

## Data Availability

The datasets generated and/or analyzed during the current study are available from the corresponding author on reasonable request.

## Supplementary Materials

The following supporting information can be downloaded at: https://www.mdpi.com/article/10.3390/jcm14061911/s1.

## References

[1] Tzotzas T., Krassas G.E. (2004). Prevalence and trends of obesity in children and adults of South Europe. Pediatr. Endocrinol. Rev..

[2] Flynn J.T., Falkner B.E. (2011). Obesity hypertension in adolescents: Epidemiology, evaluation, and management. J. Clin. Hypertens..

[3] Kelly A.S., Armstrong S.C., Michalsky M.P., Fox C.K. (2024). Obesity in Adolescents: A Review. JAMA.

[4] Lurbe E., Agabiti-Rosei E., Cruickshank J.K., Dominiczak A., Erdine S., Hirth A., Invitti C., Litwin M., Mancia G., Pall D. (2016). 2016 European Society of Hypertension guidelines for the management of high blood pressure in children and adolescents. J. Hypertens..

[5] Flynn J.T., Kaelber D.C., Baker-Smith C.M., Blowey D., Carroll A.E., Daniels S.R., De Ferranti S.D., Dionne J.M., Falkner B., Flinn S.K. (2017). Clinical Practice Guideline for Screening and Management of High Blood Pressure in Children and Adolescents. Pediatrics.

[6] Battistoni A., Canichella F., Pignatelli G., Ferrucci A., Tocci G., Volpe M. (2015). Hypertension in Young People: Epidemiology, Diagnostic Assessment and Therapeutic Approach. High Blood Press. Cardiovasc. Prev..

[7] De Onis M., Onyango A.W., Borghi E., Siyam A., Nishida C., Siekmann J. (2007). Development of a WHO growth reference for school-aged children and adolescents. Bull. World Health Organ..

[8] Giuliana G.V., Saggese G., Maffeis C. (2017). Diagnosi, trattamento e prevenzione dell’obesità del bambino e dell’adolescente. Area Pediatr..

[9] Whelton P.K., Carey R.M., Aronow W.S., Casey D.E., Collins K.J., Dennison Himmelfarb C., DePalma S.M., Gidding S., Jamerson K.A., Jones D.W. (2018). 2017 ACC/AHA/AAPA/ABC/ACPM/AGS/APhA/ASH/ASPC/NMA/PCNA Guideline for the Prevention, Detection, Evaluation, and Management of High Blood Pressure in Adults: A Report of the American College of Cardiology/American Heart Association Task Force on Clinical Practice Guidelines. Hypertension.

[10] Mancia G., Fagard R., Narkiewicz K., Redon J., Zanchetti A., Böhm M., Christiaens T., Cifkova R., De Backer G., Dominiczak A. (2013). 2013 ESH/ESC guidelines for the management of arterial hypertension: The Task Force for the Management of Arterial Hypertension of the European Society of Hypertension (ESH) and of the European Society of Cardiology (ESC). Eur. Heart J..

[11] Goulas I., Farmakis I., Doundoulakis I., Antza C., Kollios K., Economou M., Kotsis V., Stabouli S. (2021). Comparison of the 2017 American Academy of Pediatrics with the fourth report and the 2016 European Society of Hypertension guidelines for the diagnosis of hypertension and the detection of left ventricular hypertrophy in children and adolescents: A systematic review and meta-analysis. J. Hypertens..

[12] Di Bonito P., Valerio G., Pacifico L., Chiesa C., Invitti C., Morandi A., Licenziati M.R., Manco M., del Giudice E.M., Baroni M.G. (2019). Impact of the 2017 blood pressure guidelines by the American Academy of Pediatrics in overweight/obese youth. J. Hypertens..

[13] Lurbe E., Torró I., Álvarez J., Aguilar F., Mancia G., Redon J., Redon P. (2019). Impact of ESH and AAP hypertension guidelines for children and adolescents on office and ambulatory blood pressure-based classifications. J. Hypertens..

[14] Fanelli E., Di Monaco S., Pappaccogli M., Eula E., Fasano C., Masera G., Rabbone I., Rabbia F., Veglio F. (2021). Impact of 2017 AAP and 2016 ESH guidelines on paediatric hypertension prevalence. J. Hypertens..

[15] Aksoy G.K., Yapar D., Koyun N.S., Doğan Ç.S. (2021). Comparison of ESHG_2016_ and AAP_2017_ hypertension guidelines in adolescents between the ages of 13 and 16: Effect of body mass index on guidelines. Cardiol. Young.

[16] Lissau I., Overpeck M.D., Ruan W.J., Due P., Holstein B.E., Hediger M.L. (2004). Body mass index and overweight in adolescents in 13 European countries, Israel, and the United States. Arch. Pediatr. Adolesc. Med..

[17] Kvaavik E., Tell G.S., Klepp K.I. (2003). Predictors and tracking of body mass index from adolescence into adulthood: Follow-up of 18 to 20 years in the Oslo Youth Study. Arch. Pediatr. Adolesc. Med..

[18] Rudolf M.C.J., Greenwood D.C., Cole T.J., Levine R., Sahota P., Walker J., Holland P., Cade J., Truscott J. (2004). Rising obesity and expanding waistlines in schoolchildren: A cohort study. Arch. Dis. Child..

[19] Bowman S.A., Gortmaker S.L., Ebbeling C.B., Pereira M.A., Ludwig D.S. (2004). Effects of fast-food consumption on energy intake and diet quality among children in a national household survey. Pediatrics.

[20] American Academy of Pediatrics Medical Home Initiatives for Children With Special Needs Project Advisory Committee (2004). Policy statement: Organizational principles to guide and define the child health care system and/or improve the health of all children. Pediatrics.

[21] Wiehe S., Lynch H., Park K. (2004). Sugar high: The marketing of soft drinks to America’s schoolchildren. Arch. Pediatr. Adolesc. Med..

[22] Muntner P., He J., Cutler J.A., Wildman R.P., Whelton P.K. (2004). Trends in blood pressure among children and adolescents. JAMA.

[23] Flynn J.T., Alderman M.H. (2005). Characteristics of children with primary hypertension seen at a referral center. Pediatr. Nephrol..

[24] Sorof J.M., Turner J., Martin D.S., Garcia K., Garami Z., Alexandrov A.V., Wan F., Portman R.J. (2004). Cardiovascular risk factors and sequelae in hypertensive children identified by referral versus school-based screening. Hypertension.

[25] Daniels S.R. (1999). Is there an epidemic of cardiovascular disease on the horizon?. J. Pediatr..

[26] Wing Y.K., Hui S.H., Pak W.M., Ho C.K., Cheung A., Li A.M., Fok T.F. (2003). A controlled study of sleep related disordered breathing in obese children. Arch. Dis. Child..

[27] Guilleminault C., Li K., Khramtsov A., Palombini L., Pelayo R. (2004). Breathing patterns in prepubertal children with sleep-related breathing disorders. Arch. Pediatr. Adolesc. Med..

[28] Buscemi S., Giordano C. (2017). Physical activity and cardiovascular prevention: Is healthy urban living a possible reality or utopia?. Eur. J. Intern. Med..

[29] Rocchini A.P., Katch V., Kveselis D., Moorehead C., Martin M., Lampman R., Gregory M. (1989). Insulin and renal sodium retention in obese adolescents. Hypertension.

[30] Reaven G.M., Chang H., Hoffman B.B., Azhar S. (1989). Resistance to insulin-stimulated glucose uptake in adipocytes isolated from spontaneously hypertensive rats. Diabetes.

[31] Freedman D.S., Dietz W.H., Srinivasan S.R., Berenson G.S. (1999). The relation of overweight to cardiovascular risk factors among children and adolescents: The bogalusa heart study. Pediatrics.

[32] Reaven G.M., Ho H., Hoffmann B.B. (1989). Somatostatin inhibition of fructose-induced hypertension. Hypertension.

[33] Funahashi T., Matsuzawa Y. (2007). Metabolic syndrome: Clinical concept and molecular basis. Ann. Med..

[34] Kishi S., Teixido-Tura G., Ning H., Venkatesh B.A., Wu C., Almeida A., Choi E.-Y., Gjesdal O., Jacobs D.R., Schreiner P.J. (2015). Cumulative Blood Pressure in Early Adulthood and Cardiac Dysfunction in Middle Age: The CARDIA Study. J. Am. Coll. Cardiol..

[35] Aatola H., Magnussen C.G., Koivistoinen T., Hutri-Kähönen N., Juonala M., Viikari J.S., Lehtimäki T., Raitakari O.T., Kähönen M. (2013). Simplified definitions of elevated pediatric blood pressure and high adult arterial stiffness. Pediatrics.

[36] Fayyaz K., Bataineh M.F., Ali H.I., Al-Nawaiseh A.M., Al-Rifai’ R.H., Shahbaz H.M. (2024). Validity of Measured vs. Self-Reported Weight and Height and Practical Considerations for Enhancing Reliability in Clinical and Epidemiological Studies: A Systematic Review. Nutrients.

