# Supplementary material for: Prevalence of Hypertension in Adolescents: Differences Between 2016 ESH and 2017 AAP Guidelines
Source: J Clin Med. 2025 Mar 12;14(6):1911. doi: 10.3390/jcm14061911 (PMC11943055; doi:10.3390/jcm14061911)
Supplement: Supplementary file 1 [file jcm-14-01911-s001.zip › File S1.pdf]

D21) Consumi più spesso carne bianca (pollo, tacchino, coniglio) che carne rossa (manzo, maiale, hamburger, affettati, salsicce …)? □ Si □ No

D22) Quante volte alla settimana consumi verdure, pasta o riso condite con la salsa di pomodoro e il soffritto di olio, aglio, cipolla? □ 0-1 □ 2 o +

D23) Consideri la Tua alimentazione corretta?
□ SI □ NO

D24) Quanti litri di acqua bevi al giorno?

Meno di 1 litro	□
1-2 litri	□
Più di 2litri	□

D25) Quante tazzine di caffè bevi al giorno?
□ <1 □ 1-2 □ 3-5 □ 6 o +

D26) Quante volte alla settimana consumi …

	Mai	1-3 volte al mese	1-3 volte a settimana	3-5 volte a settimana	tutti i giorni o quasi
Bevande tipo cola	□	□	□	□	□
Energy drink	□	□	□	□	□

E1) Utilizzi il computer e/o il tablet? □ SI □ NO

E2) Se utilizzi il computer e/o il tablet, per quante ore al giorno?
□ 1-2 ore □ 3-5 ore □ più di 5 ore

E3) Che tipo di uso pensi di fare del computer e/o del tablet?

Eccessivo	□
Moderato	□
Scarso	□

E5) Riusciresti a fare a meno del computer e/o del tablet? □ SI □ NO

E6) La Tua vita senza il computer e/o il tablet sarebbe…
(possibile dare più di una risposta)

Migliore	□
Peggiore	□
Possibile	□
Impossibile	□

E7) Scrivi tre parole che indichino lo stato d'animo che provi quando usi il computer e/o il tablet.

____________ ____________ ____________

E8) Possiedi uno smartphone e/o similari? □ SI □ NO

E9) Che tipo di uso pensi di fare dello smartphone e/o similari?

Eccessivo	□
Moderato	□
Scarso	□
