# Supplementary material for: Prevalence of Hypertension in Adolescents: Differences Between 2016 ESH and 2017 AAP Guidelines
Source: J Clin Med. 2025 Mar 12;14(6):1911. doi: 10.3390/jcm14061911 (PMC11943055; doi:10.3390/jcm14061911)
Supplement: Supplementary file 1 [file jcm-14-01911-s001.zip › File S2.pdf]

E10) Riusciresti a fare a meno dello smartphone e/o similari? □ SI □ NO

E11) La Tua vita senza lo smartphone e/o similari sarebbe…
(possibile dare più di una risposta)

Migliore	□
Peggiore	□
Possibile	□
Impossibile	□

E12) Scrivi tre parole che indichino lo stato d'animo che provi quando usi smartphone e/o similari.

____________ ____________ ____________

E13) Hai bevuto alcolici negli ultimi 12 mesi? □ SI □ NO

E14) Se si, con quale frequenza?
□1 volta al mese o meno □2-4 volte al mese □2-3 volte a settimana □4-5 volte a settimana

□6 o più volte a settimana

E15) Negli ultimi 12 mesi, quanti drink hai assunto in un giorno tipico in cui ha bevuto?
Per "drink" si intende un bicchiere di vino (circa 15 cl), una bottiglia/lattina di birra (33 cl), un bicchierino di liquore (5 cl) o un cocktail).
□ 0 drink □ 1-2 drink □ 3-4 drink □ 5-6 drink □ 7-9 drink □ 10 o più drink

E16) Negli ultimi 12 mesi, ti è capitato di bere 6 o più drink in una singola occasione?
□ mai □ meno di 1 volta al mese □1-3 volte al mese □1-3 volte a settimana □ tutti i giorni o quasi

E17) Hai bevuto alcolici nell'ultimo mese? □ Si □ No

E18) Se sì, in quante occasioni hai bevuto uno o più dei seguenti alcolici negli ultimi giorni?

	1-3 volte al mese	1 volta a settimana	2-3 volte a settimana	4-5 volte a settimana	Quasi tutti i giorni
Birra	□	□	□	□	□
Vino	□	□	□	□	□
Aperitivi alcolici	□	□	□	□	□
Drink leggeri	□	□	□	□	□
Liquori / superalcolici	□	□	□	□	□
Mix di energy drink e alcol	□	□	□	□	□

E19) In quali momenti della giornata assumi bevande alcoliche?
(possibile dare più di una risposta)

Solo ai pasti	□
Prima di cena (aperitivo)	□
Dopo cena	□
In qualsiasi momento della giornata	□

E20) Hai mai pensato che il tuo consumo di alcol fosse eccessivo? □ SI □ NO

E21) Fumi? □ SI □ NO

E22) Se fumi, quante sigarette al giorno?

Meno di 5 sigarette	□
Da 5 a 20 sigarette	□
Da 21 a 40 sigarette	□
Più di 40 sigarette	□

E23) Utilizzi sigarette elettroniche o altri dispositivi analoghi? □ SI □ NO

E24) Se fumi, ha mai cercato di smettere? □ SI □ NO

E25) Se non fumi, ha mai fumato in passato? □ SI □ NO

E26) Se non fumi, sei esposto a fumo passivo? □ SI □ NO

E27) Hai mai fatto uso di droghe almeno una volta in vita tua? □ SI □ NO

E28) Se sì, quale /i? *(possibile più di una risposta)*
□ Cannabis (Hashish e/o marijuana) □ Cocaina □ Crack □ Anfetamine □ Ecstasy / MDMA
□Eroina e altri oppiacei (oppio, morfina, metadone) □ Allucinogeni (LSD, funghi, ketamina)
□ Sostante solventi/inalanti (colle, gas, popper)

E29) In quante occasioni, se ce ne sono state, ha usato le seguenti sostanze negli ultimi 12 mesi?
(possibile dare più di una risposta)

	Mai	1-2	3-5	6-9	10-19	20-39	40 o più
Cannabis (Hashish e/o marijuana	□	□	□	□	□	□	□
Cocaina	□	□	□	□	□	□	□
Crack	□	□	□	□	□	□	□
Anfetamine	□	□	□	□	□	□	□
Ecstasy / MDMA	□	□	□	□	□	□	□
Eroina e altri oppiacei (oppio, morfina, metadone)	□	□	□	□	□	□	□
Allucinogeni (LSD, funghi, ketamina)	□	□	□	□	□	□	□
Sostante solventi/inalanti (colle, gas, popper)	□	□	□	□	□	□	□

E30) In quante occasioni, se ce ne sono state, ha usato le seguenti sostanze negli ultimi 30 giorni?
(possibile dare più di una risposta)

	Mai	1-2	3-5	6-9	10-19	20-39	40 o più
Cannabis (Hashish e/o marijuana	□	□	□	□	□	□	□
Cocaina	□	□	□	□	□	□	□
Crack	□	□	□	□	□	□	□
Anfetamine	□	□	□	□	□	□	□
Ecstasy / MDMA	□	□	□	□	□	□	□
Eroina e altri oppiacei (oppio, morfina, metadone)	□	□	□	□	□	□	□
Allucinogeni (LSD, funghi, ketamina)	□	□	□	□	□	□	□

Sostante solventi/inalanti (colle, gas, popper)	□	□	□	□	□	□	□

E31) Quanto dista la Tua abitazione dalla scuola?

Meno di 5 Km □
Tra 5 Km e i 15 Km □
Più di 15 Km □
Altro (specificare_________________)

E32) Quale mezzo utilizzi per recarti a scuola? *(possibile dare più di una risposta)*

Non uso nessun mezzo, vado a piedi □
Bicicletta □
Moto o scooter □
Auto □
Mezzo pubblico (autobus, metropolitana, treno) □
Altro (specificare________________________)

E34) Pratichi regolarmente attività fisica?

□ SI □ NO

E35) Se pratichi attività fisica, che tipo di attività svolgi? *(possibile dare più di una risposta)*

Corsa □
Passeggiate all'aria aperta □
Calcio o calcetto □
Palestra □
Piscina □
Bicicletta □
Altro (specificare________________________)

E36**) Se pratichi attività fisica, quanti giorni a settimana?**

Tutti i giorni della settimana □
Alcuni giorni *(indicare il numero_____)* □
Solo il fine settimana □

E37) Se svolgi attività fisica, per quanto tempo la pratichi ogni volta?

Meno di un'ora □
1 ora □
2 ore □
più di 2 ore □

E38) Se svolgi attività fisica, la pratichi a livello…

Amatoriale □
Agonistico □

E39) Secondo Te fai abbastanza movimento durante il giorno?

□ SI □ NO
